# Epigenetic histone modification by butyrate downregulates KIT and attenuates mast cell function

**DOI:** 10.1111/jcmm.17924

**Published:** 2023-08-21

**Authors:** Ravindra Gudneppanavar, Emma Elizabeth Sabu Kattuman, Lakshminarayan Reddy Teegala, Erik Southard, Ramakumar Tummala, Bina Joe, Charles K. Thodeti, Sailaja Paruchuri

**Affiliations:** ^1^ Department of Physiology and Pharmacology The University of Toledo College of Medicine and Life Sciences Toledo Ohio USA

**Keywords:** asthma, butyrate, HDAC, KIT, MAPK, MC, proliferation, SCF, viability

## Abstract

Short‐chain fatty acid butyrate is produced from the bacterial fermentation of indigestible fiber in the intestinal lumen, and it has been shown to attenuate lung inflammation in murine asthma models. Mast cells (MCs) are initiators of inflammatory response to allergens, and they play an important role in asthma. MC survival and proliferation is regulated by its growth factor stem cell factor (SCF), which acts through the receptor, KIT. It has previously been shown that butyrate attenuates the activation of MCs by allergen stimulation. However, how butyrate mechanistically influences SCF signalling to impact MC function remains unknown. Here, we report that butyrate treatment triggered the modification of MC histones via butyrylation and acetylation, and inhibition of histone deacetylase (HDAC) activity. Further, butyrate treatment caused downregulation of SCF receptor KIT and associated phosphorylation, leading to significant attenuation of SCF‐mediated MC proliferation, and pro‐inflammatory cytokine secretion. Mechanistically, butyrate inhibited MC function by suppressing KIT and downstream p38 and Erk phosphorylation, and it mediated these effects via modification of histones, acting as an HDAC inhibitor and not via its traditional GPR41 (FFAR3) or GPR43 (FFAR2) butyrate receptors. In agreement, the pharmacological inhibition of Class I HDAC (HDAC1/3) mirrored butyrate's effects, suggesting that butyrate impacts MC function by HDAC1/3 inhibition. Taken together, butyrate epigenetically modifies histones and downregulates the SCF/KIT/p38/Erk signalling axis, leading to the attenuation of MC function, validating its ability to suppress MC‐mediated inflammation. Therefore, butyrate supplementations could offer a potential treatment strategy for allergy and asthma via epigenetic alterations in MCs.

## 1 INTRODUCTION

Mast cells (MCs) are important first responders of the immune system that are distributed at interfaces of the external environment.^1^ They initiate inflammatory responses to allergens and infectious agents by secreting several inflammatory mediators.^2^, ^3^, ^4^ MC maturity, survival and proliferation are regulated by stem cell factor (SCF), via its tyrosine kinase receptor, KIT.^3^ Upon ligand binding and receptor dimerisation, the phosphorylation of tyrosine residues within the KIT receptor triggers intracellular signalling pathways to regulate cell survival, proliferation, mediator release and migration.^3^ We and others have previously shown that SCF stimulation induces the phosphorylation of p38 and Erk mitogen activated protein kinases (MAPK) in murine bone marrow‐derived MC (BMMCs).^5^, ^6^ MAPKs integrate multiple signals from various receptors and second messengers, and they are involved in the regulation of cellular proliferation and differentiation.^7^ It has previously been shown that the airways of asthmatic patients have increased SCF expression,^8^ which induces MC hyperplasia. Further, increased levels of MC mediators were detected in the bronchoalveolar lavage (BAL) fluid of asthmatic patients.^9^

Short chain fatty acids (SCFA; acetate, propionate and butyrate) are metabolites generated from the bacterial fermentation of indigestible fiber and aminoacids.^10^ Butyrate is mainly produced by members of Firmicutes phylum, including the genera *Roseburia*, *Pseudobutyrivibrio* and *Faecalibacterium*, among others.^11^ Butyrate producers in the intestine or enhanced butyrate levels negatively correlate with asthma severity in both murine and human studies. High levels of butyrate in early age (~1 year) have been shown to confer protection against atopy at a later age (~6 year).^12^ Accordingly, in a murine ovalbumin (OVA) model of allergic inflammation, mice fed with butyrate exhibited attenuated inflammation via T cell‐ and dendritic cell‐dependent mechanisms.^13^ Notably, antibiotic treatment during early ages that deplete gut microbiota, was shown to augment susceptibility to asthma.^14^ All these studies suggest that butyrate generated in the gut or butyrate supplementation can help alleviate asthma severity. Butyrate relays signalling mainly through three G‐protein‐coupled receptors, GPR41/FFAR3, GPR43/FFAR2 and GPR109A/HCAR2, and the affinity for these receptors is in the high μM to low mM range.^10^ Apart from these traditional receptors, butyrate was shown to signal via peroxisome proliferator‐activated receptor gamma,^15^ or by acting as a potent histone deacetylase (HDAC) inhibitor.^10^, ^15^ HDACs regulate gene expression by removing acetyl groups on specific lysine residues from histone and non‐histone proteins, thereby modulating chromatin structure. Although SCFA have been linked to asthma and allergy, limited studies focused on their role in MCs, the initiators of allergic inflammation. Even these studies focused on the role of butyrate in FcεR1‐mediated MC activation and associated inflammation. The mechanism by which butyrate regulates inflammatory/proliferatory signals downstream of SCF in primary MCs is not known. Therefore, in the current study, we investigated the mechanistic aspects of how butyrate impacts the SCF/KIT signalling axis and associated MC function.

## 2 MATERIALS AND METHODS

### 2.1 Animals

Wild type C57BL/6 (8–12 weeks old) mice were used to culture the bone marrow‐derived mast cells (BMMCs). The animals were purchased from Jackson Laboratories and were maintained at the department of laboratory animal resources (DLAR) at the University of Toledo Medical Center (UTMC). Animals were euthanized in accordance with standard guidelines as reviewed and approved by the Institutional Animal Care and Use Committee of UTMC.

### 2.2 Reagents

The following chemicals and reagents were commercially purchased: SCF (Peprotech), Sodium acetate, Sodium butyrate, Sodium propionate (EMD Millipore Corporation), SAHA (pan‐ HDAC inhibitor) and SBHA (HDAC 1 and 3 inhibitor), BIRB0796 (p38 MAPK inhibitor), PD98059 (MEK inhibitor) (Tocris Bioscience). Primary antibodies phospho‐KIT (Y719), phospho‐p38 (T180/Y182), phospho‐Erk (T202/Y204), Total KIT, p38 and Erk from Cell Signalling Technology, GAPDH (Fitzgerald), secondary antibodies anti‐rabbit and anti‐mouse (Jackson ImmunoResearch), PE‐Conjugated rat anti‐mouse CD117 (KIT) antibody (Biolegend). MIP1β, MCP‐1 and TNFα ELISA kits from R&D Systems. Transcriptor first stand cDNA synthesis kit and light cycler 480 SYBR Green I Master Mix (Roche), XTT viability assay kit (R&D Systems), BrdU proliferation assay kit (EMD Millipore Corporation), EpiQuik Nuclear Extraction kit, EpiQuik Total Histone Extraction kit and EpiQuik HDAC Activity/Inhibition Assay Kit (Colorimetric) are from EPIGENTEK.

### 2.3 Cell culture

Bone marrow cells (BMCs) were isolated from the tibia and femur bones of wild type (WT) C57BL/6 mice. Cells were re‐suspended in RPMI‐1640 medium supplemented with 10% FBS, 1% penicillin–streptomycin, L‐glutamine (2 mM), sodium pyruvate (1 mM), HEPES buffer (25 mM), and β‐mercaptoethanol (50 μM); were maintained at 37°C with 5% CO_2_ and 95% humidity. BMCs were differentiated into BMMCs using 30 ng/mL of mouse interleukin 3 (IL‐3) for 5 weeks. The maturity of BMMCs was examined by toluidine blue staining, and >90% mature BMMCs were used for experiments. Differentiation of BMCs into BMMCs was confirmed by analysing the surface expression of KIT and FcεR1 via flow cytometry.^5^

### 2.4 Cell activation and treatment

For cell proliferation and viability assays, BMMCs were stimulated with SCF (100 ng/mL) with or without sodium butyrate (0.1–10 mM), β‐hydroxy butyrate (BHB; 5 mM), GLPG (0.1 μM) SBHA (20 μM) and SAHA (1 μM) in the absence of IL‐3 for 72 h. To analyse the protein expression of KIT and the post translational modification of histones by butyrate, BMMCs were treated with sodium butyrate as described earlier in literature for 24 h.^15^ In some experiments, cells were pretreated with SBHA (20 μM), and SAHA (1 μM) for 30 min, followed by SCF treatment. For transcript and chemokine analysis, BMMCs were treated with butyrate (24 h) and stimulated with SCF (100 ng/mL) in the absence of IL‐3 for 4 h. For the analysis of signalling intermediates, BMMCs were stimulated with SCF (100 ng/mL) in the absence of IL‐3 for 30 min after butyrate treatment (24 h).

### 2.5 Total histone extraction

BMMCs (1 × 10^6^) were treated with sodium butyrate (5 mM) for 24 h, and histones were extracted using an EpiQuik Total Histone extraction kit, according to the manufacturer's protocol. Protein concentration was quantified by BCA assay, and the extracts were stored at −80°C.

### 2.6 Nuclear extraction and HDAC activation assay

Nuclear extraction was performed using an EpiQuik Nuclear Extraction Kit, according to the manufacturer's protocol. Nuclear protein concentration was quantified by BCA assay and further used to analyse HDAC activity. HDAC activity was analysed using an EpiQuik HDAC Activity/Inhibition Assay Kit (Colorimetric), according to manufacturer's protocol, with 2 μg of nuclear protein extract. HDAC activity was measured calorimetrically by measuring the absorbance at 450 nm using Microplate reader.

### 2.7 Cell lysates and western blotting

Following stimulations, BMMCs (0.5 × 10^6^) were lysed with lysis buffer (BD Bioscience) supplemented with Protease and Phosphatase Inhibitor Cocktails (Thermo fisher scientific). Immunoblotting was performed as described previously.^16^ Nuclear extracts or cell lysates were subjected to 4%–15% SDS‐PAGE and transferred to PVDF membrane. The membranes were blocked for 1 h with 5% non‐fat dried milk in 1x TBS/ 0.1% Tween‐20 at room temperature, and then were subsequently incubated with respective primary phospho‐ antibodies diluted in 5% non‐fat dried milk/ 1x TBS/ 0.1% Tween‐20 (1:1000) overnight at 4°C on shaker. The next day, the membranes were washed three times with 1x TBS/ 0.1% Tween‐20 and then incubated with the secondary antibody (peroxidase‐conjugated anti‐rabbit) (1:5000) for 1 h at room temperature. Thereafter, the membranes were washed again, incubated with ECL, and the bands were visualized using ProteinSimple imager and quantified using Image J. Densitometric analysis was performed by normalising the respective bands to loading control.

### 2.8 Flow cytometry

Following treatment with sodium butyrate as mentioned above, BMMCs (1 × 10^5^) were washed with FACS buffer (0.5% bovine serum albumin in phosphate buffered saline), fixed with 4% paraformaldehyde, permeabilized, and incubated with purified PE‐conjugated rat anti‐mouse KIT antibodies (Biolegend, San Diego, CA) for 30 min. The cells were washed with FACS buffer three times, and flow cytometric analyses were performed using BD Accuri C6 Plus Flow Cytometer.

### 2.9 Real‐time quantitative PCR (qPCR)

The expressions of inflammatory transcripts were determined with qPCR performed on Light cycler 480 (Roche).^17^ Following indicated treatments, the total RNA was isolated with an E.Z.N.A. Total RNA kit 1 (Omega Bio‐Tek). DNAse contamination was removed using a RNAse‐free DNA Removal Kit (Omega Bio‐Tek) based on the manufacturer's instructions. cDNA was synthesized using a cDNA synthesis kit (Roche). qPCR was performed using the primers mentioned below. The levels of respective genes relative to the GAPDH were analysed, and the ∆ΔCT values were calculated and expressed as relative expression or fold change compared to control (no template). The quality of the RNA, primers and qPCR reaction was validated using proper controls, like no RT control or no template control.

Primers.

mMIP1β‐ F: 5’‐TCTCTCTCCTCTTGCTCGT‐3′; R: 5’‐CCGGGAGGTGTAAGAGAAAC‐3’

mMCP‐1‐ F: 5’‐AGTAGGCTGGAGAGCTACAA‐3′; R: 5’‐GTATGTCTGGACCCATTCCTT‐3’

mTNFα‐ F: 5’‐CTATGTCTCAGCCTCTTCTCATTC‐3′; R: 5’‐GAGGCCATTTGGGAACTTCT‐3’

mCOX‐2‐ F: 5’‐AACCGCATTGCCTCTGAAT‐3′; R: 5’‐CATGTTCCAGGAGGATGGAG‐3’.

mCOX‐1‐ F: 5’‐GGATACTGGCTCTGGGAATTTG‐3′; R: 5’‐GTAGTCATGCGCTGAGTTGTAG‐3’

mGAPDH‐ F: 5’‐CTCCCACTCTTCCACCTTCG‐3′; R: 5’‐CCACCACCCTGTTGCTGTAG‐3’

### 2.10 Cell viability and proliferation‐

Proliferation and viability assays were performed in triplicates on cells plated at a density of (1 × 10^4^), as mentioned earlier.^16^, ^18^ Cells were treated in the presence or absence of SCF (100 ng/mL), and pretreated or not with sodium acetate or sodium propionate or sodium butyrate (0.1–10 mM) and indicated HDAC inhibitors. Viability and proliferation were measured after 72 h by XTT and BrdU assay respectively, according to the manufacturer's protocol. XTT is a measure of metabolic activity of the cells. For the proliferation assay, BrdU label was added 24 h before the assay.

### 2.11 ELISA

The concentration of MCP‐1 and MIP1β secreted into the medium by BMMCs after respective treatments were analysed by MCP‐1 ELISA kit, MIP1β ELISA kit and TNFα ELISA kit (all from R & D Systems) according to the manufacturer's protocol.^17^

### 2.12 Statistics

Data are expressed as mean ± SEM from at least three experiments. Significance was determined using one‐way anova, and comparison between the groups were determined by Tukey's multiple comparisons test (GraphPad Prism, 7.01). *p < 0.05, **p < 0.01, ***p < 0.001, ****p < 0.0001, NS = not significant.

## 3 RESULTS

### 3.1 Butyrate causes epigenetic modification of MC histones

**FIGURE 1:**
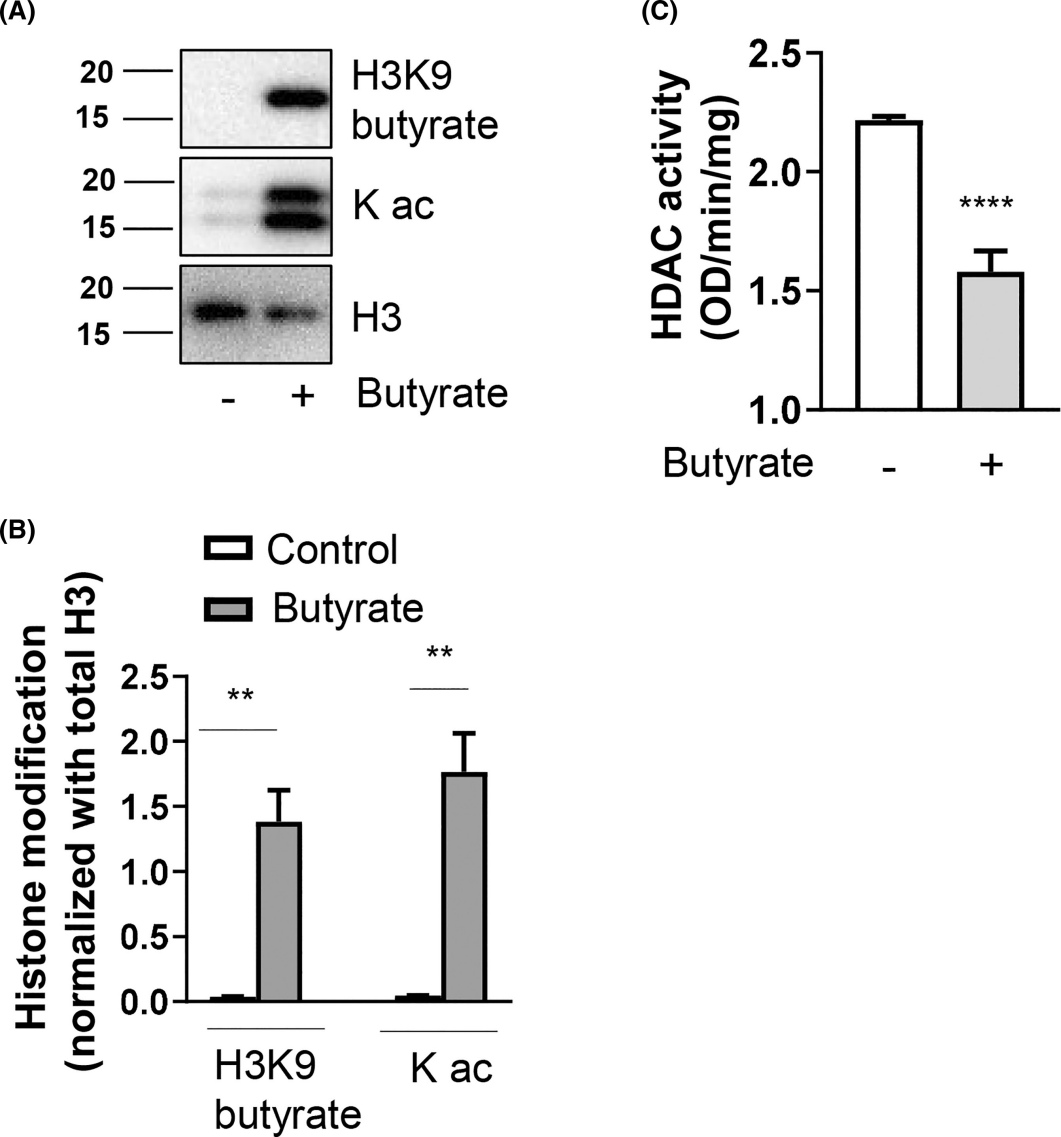
Epigenetic modification of histones by butyrate. BMCs were isolated and differentiated into BMMCs using murine interleukin‐3 (IL‐3; 30 ng/mL) for 5 weeks. BMMCs (1 × 10^6^) were treated with sodium butyrate (5 mM) for 24 h, and histones were extracted. Histone proteins were subjected to western blotting using H3K9 butyrylation, pan lysine acetylation and total histone H3 antibodies. (A) Western blot of histones using butyrylation, acetylation, and total Histone antibodies. (B) Densitometric analysis of data shown in (A). (C) Nuclear extracts were prepared from untreated and butyrate (5 mM) treated cells and 2 μg of nuclear protein was used to analyse HDAC activity. HDAC activity was measured colorimetrically by measuring the absorbance at 450 nm using Microplate reader. Data are represented as mean ± SEM of three separate experiments. The significance was tested using one‐way anova and post hoc analysis. *p ≤ 0.05, **p ≤ 0.01, ****p ≤ 0.0001, ns = not significant.

To determine how butyrate modulates MC function, BMMCs were treated with butyrate, and histone modifications were assessed in the nuclear lysates by western botting. We observed a significant butyrylation of histone H3 at lysine 9 (H3K9) by butyrate (Figure 1A,B). Apart from H3K9 butyrylation, we also observed significant histone acetylation (Figure 1A,B) suggesting that butyrate modulates MC function through its actions on histones. Although treatment with 0.5 mM butyrate caused modest H3K9 butyrylation and acetylation (not shown), higher concentrations (5 mM) are required for significant histone modifications (Figure 1A,B). Further, butyrate treatment significantly attenuated HDAC activity (Figure 1C).

### 3.2 Butyrate downregulates KIT receptor

**FIGURE 2:**
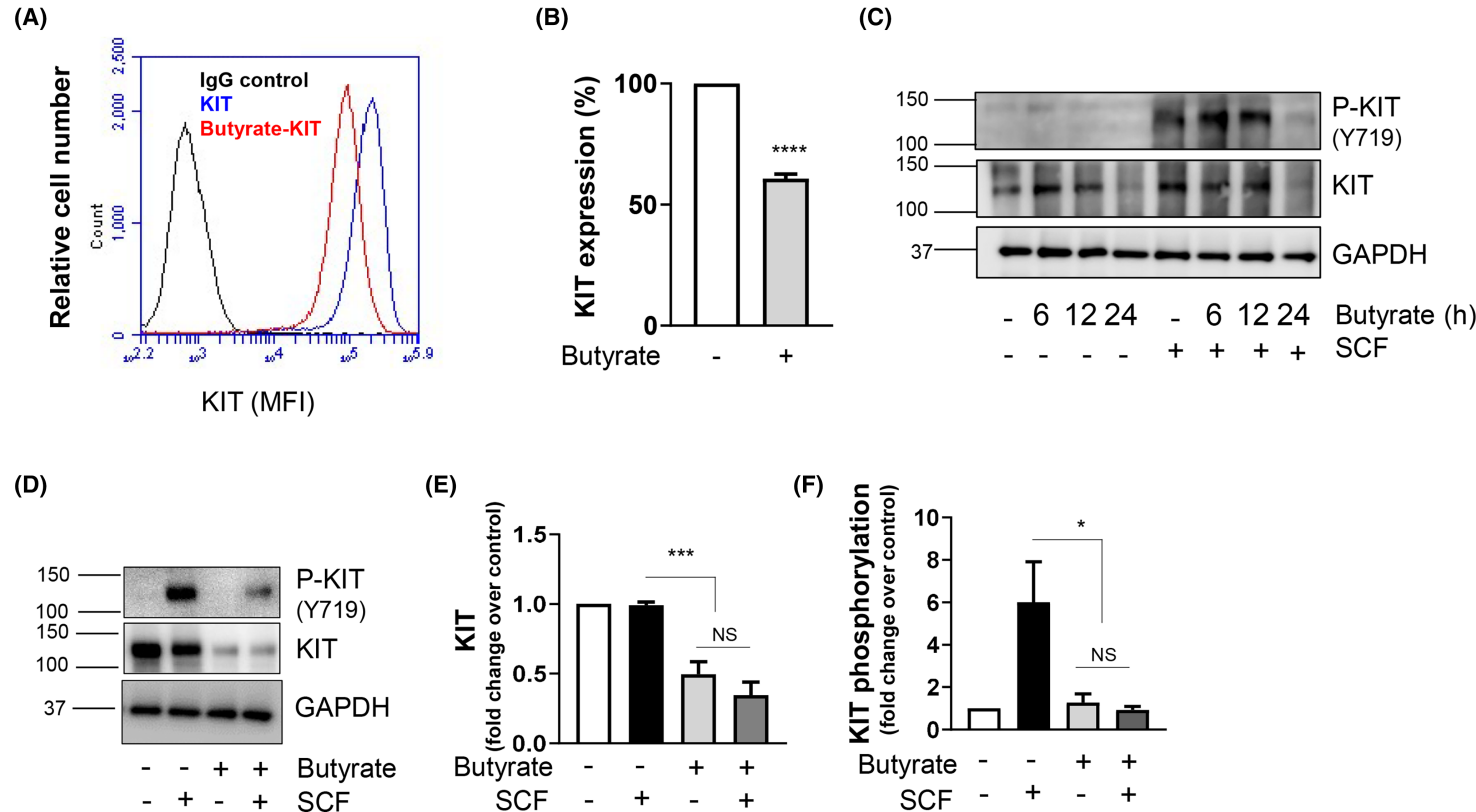
Butyrate downregulates KIT and attenuate KIT phosphorylation. (A) Effect of butyrate on internalization of KIT receptors in BMMCs. BMMCs were treated with butyrate (5 mM, 24 h) and surface expression of KIT receptor was analysed by flow cytometry. (B) Quantification of data shown in (A) and expressed as net mean fluorescence intensity (MFI). (C) BMMCs were treated with butyrate (5 mM, 6, 12 and 24 h) followed by treatment with SCF (100 ng/mL) for 30 min. KIT phosphorylation was assessed by western blotting using phospho‐specific KIT (Y719) antibody. Blots were re‐probed for total KIT and GAPDH. (D) BMMCs were treated with butyrate (5 mM, 24 h) followed by treatment with SCF (100 ng/mL) for 30 min. KIT phosphorylation was assessed by western blotting using phospho‐specific KIT (Y719) antibody. Blots were re‐probed for total KIT and GAPDH. (E) and (F) represent densitometric analysis of data shown in C. Data are represented as mean ± SEM of three separate experiments. The significance was tested using one‐way anova and post hoc analysis. *p ≤ 0.05, ***p ≤ 0.001, ****p ≤ 0.0001, ns = not significant.

Since butyrate treatment caused epigenetic modifications in MCs, we speculated that these modifications may affect MC functions. SCF is the principal growth factor for MCs^3^ and play a critical role in regulating human and BMMC function via its receptor KIT.^19^ Therefore, we analysed KIT expression in response to butyrate. Flow cytometric analysis revealed a significant left ward shift (40% reduction) in KIT expression with butyrate treatment, suggesting its downregulation (Figure 2A,B). To determine the temporal regulation of KIT by butyrate, we treated BMMCs with butyrate at 6, 12 and 24 h and analysed KIT expression. Western blot analysis confirmed a significant reduction in KIT expression and its phosphorylation only in cells exposed to butyrate for 24 h (Figure 2C–F).

### 3.3 Butyrate reduces SCF‐mediated MC viability and proliferation

**FIGURE 3:**
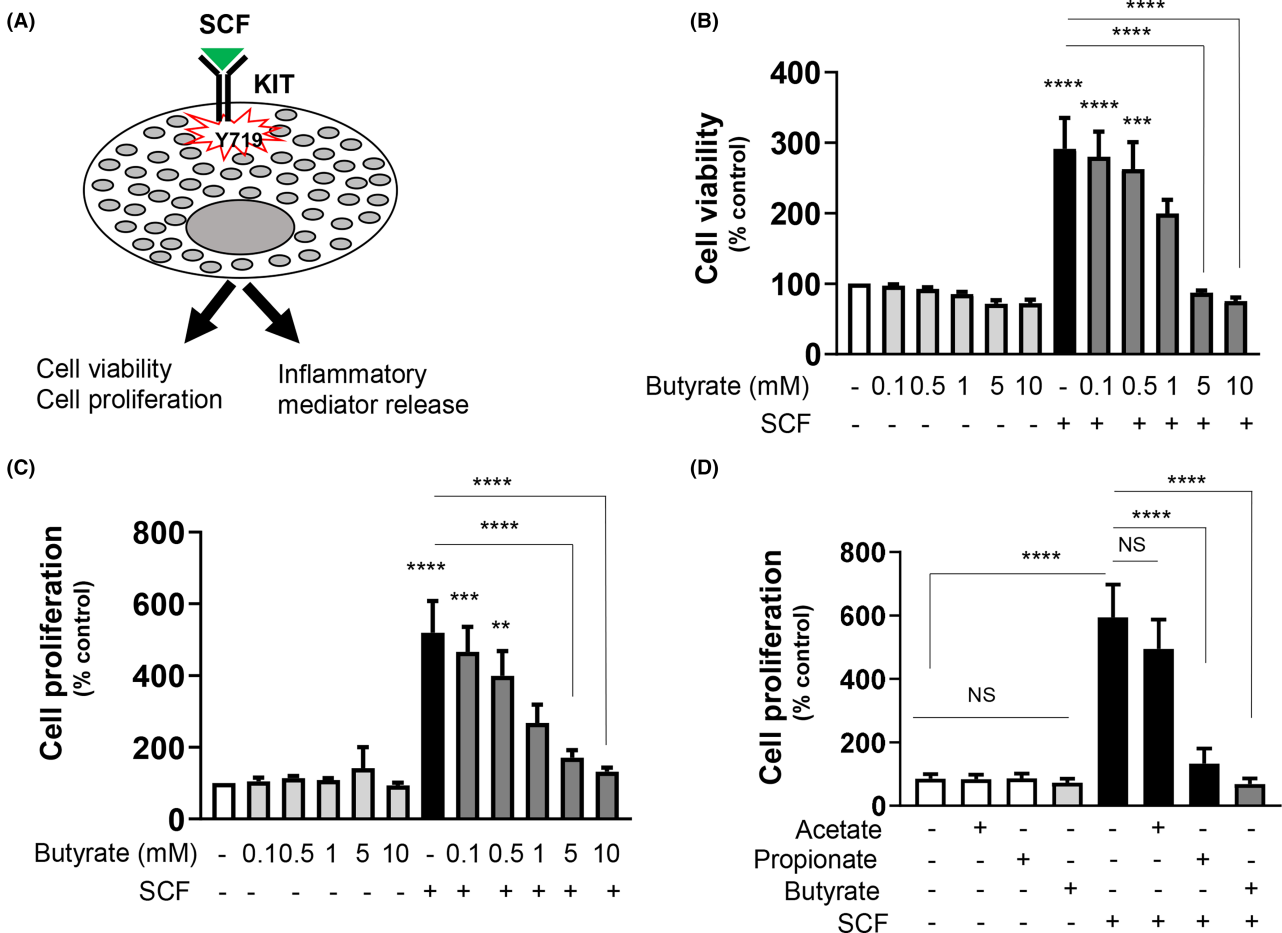
Butyrate attenuates SCF‐mediated BMMC viability and proliferation. (A). Cartoon depicting that SCF/KIT axis promotes MC activation. BMMCs were plated in triplicate at a density of (1 × 10^4^) in each well of a 96 well plate suspended in fresh medium without IL‐3 and in the presence or absence of SCF (100 ng/mL), pretreated or not with butyrate (5 mM, 24 h). (B) Cell viability was analysed by XTT assay after 72 h. (C) Cell proliferation was measured after 72 h by BrdU ELISA. BrdU label was added 24 h before the assay. (D) Cell proliferation was measured by BrdU ELISA as described above in BMMCs pretreated with 5 mM of other SCFA; acetate, propionate and butyrate. Viability and proliferation data are represented as percentage over control and mean ± SEM of three separate experiments. The significance was tested using one‐way anova and post hoc analysis. **p ≤ 0.01, ***p ≤ 0.001, ****p ≤ 0.0001, ns = not significant.

The SCF/KIT axis regulates several aspects of BMMC functions, including viability, proliferation, and inflammatory responses (Figure 3A). We further tested if butyrate can influence any of these SCF‐mediated responses. BMMCs were pretreated with butyrate (0.1–10 mM) for 24 h, followed by SCF treatment (100 ng/mL) for 72 h, and then their viability and proliferation were measured. As reported previously,^5^ SCF treatment increased both the viability and proliferation of BMMCs (Figure 3B,C). Although we observed a reduction in BMMC viability and proliferation at 1 mM butyrate, we observed a significant reduction only with 5 mM (the concentration at which butyrate caused epigenetic changes) and 10 mM. Notably, among all the SCFA, butyrate was the most effective, compared to acetate or propionate, in inhibiting SCF‐mediated proliferation (Figure 3D).

### 3.4 Butyrate attenuates SCF‐mediated pro‐inflammatory mediator expression in BMMCs

**FIGURE 4:**
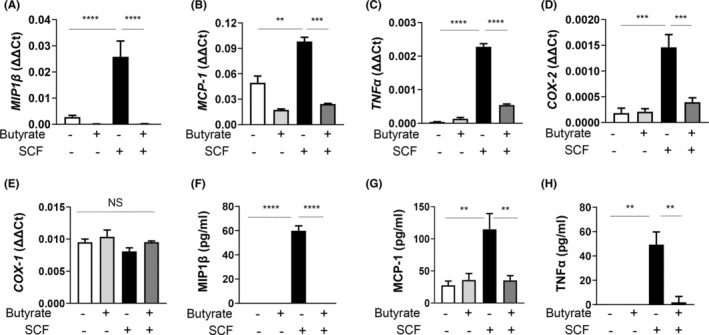
Butyrate attenuates SCF‐mediated inflammatory mediator release. BMMCs were pretreated or not with butyrate (5 mM) for 24 h, and stimulated in the presence or absence of SCF (100 ng/mL) for 4 h. RNA was extracted followed by cDNA synthesis and transcript levels of MIP1β (A), MCP‐1 (B), TNFα (C), COX‐2 (D), and COX‐1 (E) were measured by qPCR and analysed compared to GAPDH. Culture supernatants were collected and analysed for MIP1β (F), MCP‐1 (G), TNFα (H) proteins by ELISA. Data are represented as mean ± SEM of three separate experiments. The significance was tested using one‐way anova and post hoc analysis. **p ≤ 0.01, ***p ≤ 0.001, ****p ≤ 0.0001, NS = not significant.

Butyrate has been previously shown to attenuate IgE and non‐IgE‐mediated MC degranulation and IL‐6 release.^15^ Therefore, we examined whether butyrate could also impact SCF‐mediated pro‐inflammatory cytokine expression and release apart from regulating MC numbers. Similar to our previous observations,^20^ SCF upregulated MIP1β, MCP‐1, TNFα and COX‐2 transcripts. Importantly, butyrate treatment significantly inhibited all these transcripts (Figure 4A–D), without affecting COX‐1 (Figure 4E). Further, butyrate inhibited SCF‐induced MIP1β, MCP‐1 and TNFα secretion (Figure 4F–H), complementing the transcript data.

### 3.5 Butyrate attenuates SCF‐mediated phosphorylation of p38 MAPK and Erk

**FIGURE 5:**
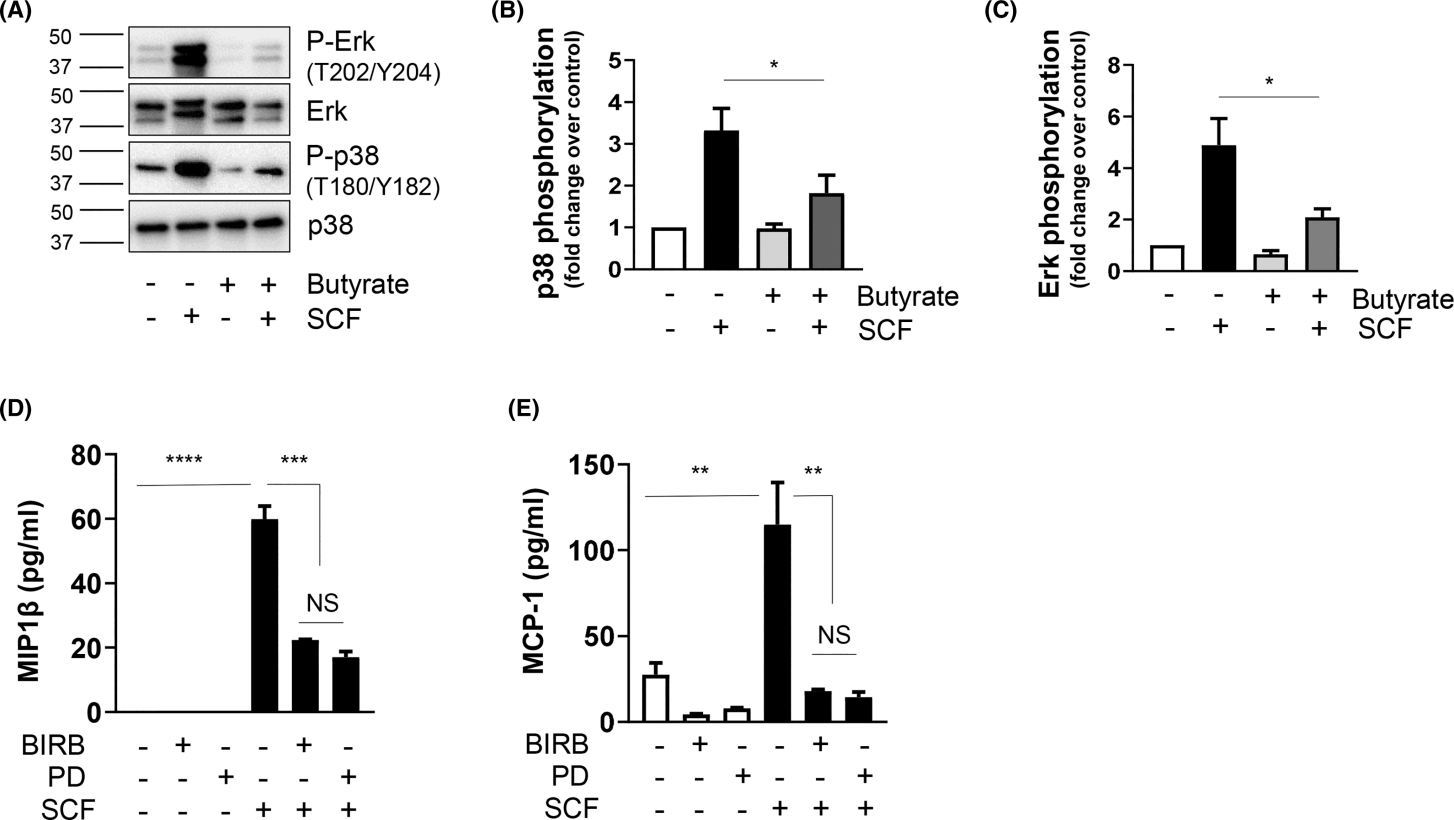
Butyrate attenuates SCF‐mediated phosphorylation of p38 and Erk‐ BMMCs were pretreated or not with butyrate (5 mM) for 24 h, and stimulated in the presence or absence of SCF (100 ng/mL) for 30 min. (A) p38 and Erk phosphorylation and expression was determined in the cell lysates by western blotting. Blots were re‐probed for total KIT and GAPDH. (B, C) represent densitometric analysis of data shown in (A). BMMCs were stimulated in the presence or absence of SCF (100 ng/mL; 4 h) with or without BIRB0796 (0.1 μM) and PD98059 (50 μM) and MIP1β (D) and MCP‐1(E) expression in the culture medium was determined by ELISA. Data are represented as mean ± SEM of three separate experiments. The significance was tested using one‐way anova and post hoc analysis. *p ≤ 0.05, **p ≤ 0.01, ***p ≤ 0.001, ****p ≤ 0.0001, NS = not significant.

SCF has been previously shown to modulate BMMC proliferation via MAPK.^5^ Therefore, we asked if the MAPK family members are also sensitive to butyrate treatment. We observed a significant reduction in phosphorylated p38 MAPK and Erk with butyrate treatment in SCF‐treated BMMCs (Figure 5A–C). Although p38 MAPK and Erk have been known to play a role in BMMC proliferation,^5^ their role in SCF‐induced mediator release is not clear. Therefore, we analysed SCF‐mediated chemokine secretion in response to a specific p38 and Erk inhibitors, BIRB0796 and PD98059 respectively. We observed a significant reduction in MIP1β and MCP‐1 secretion in response to both the inhibitors (Figure 5D,E). Together, these data suggest that butyrate regulates MC function by downregulating KIT and associated p38 and Erk phosphorylation.

### 3.6 Butyrate induces MC function independent of GPR41 or GPR43, but possibly via epigenetic modification and KIT repression via HDAC1/3

**FIGURE 6:**
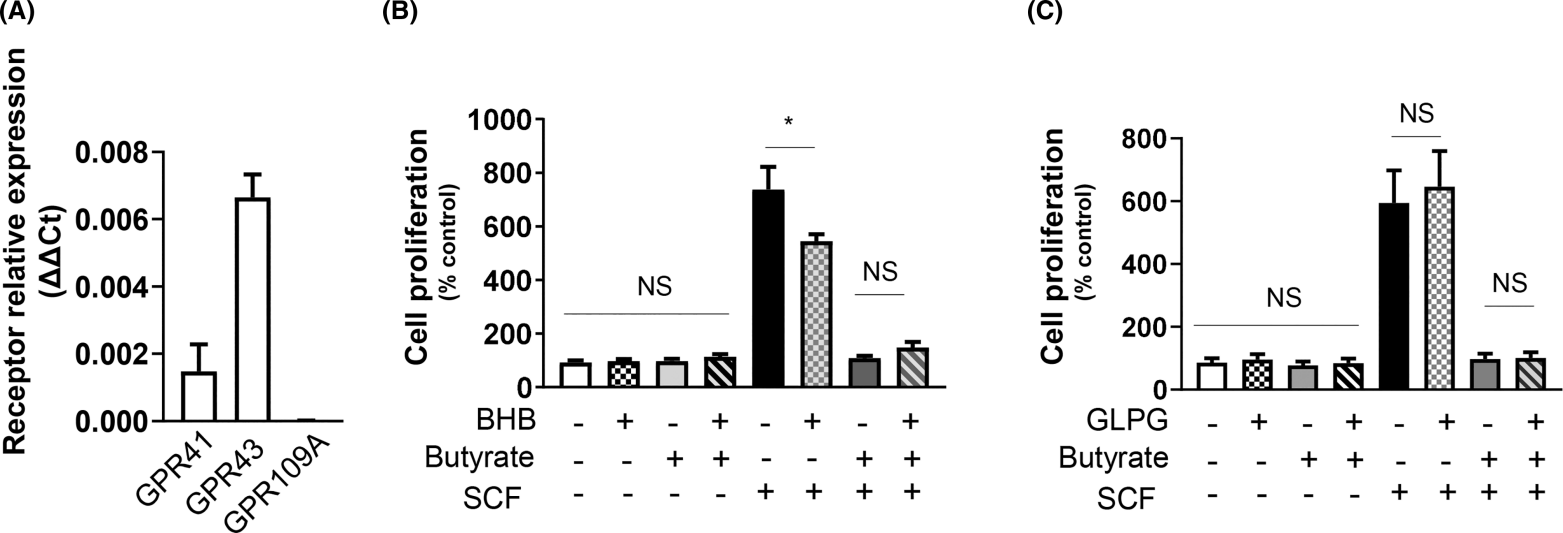
Butyrate induces MC function independent of GPR41 or GPR43. (A) BMMCs were analysed for the expression of GPR41, GPR43, and GPR109A by qPCR. BMMCs were plated in triplicate at a density of (1 × 10^4^) in each well of a 96 well plate suspended in fresh medium without IL‐3 and in the presence or absence of SCF (100 ng/mL), pretreated or not with butyrate ± BHB (both 5 mM, 24 h) (B) and butyrate ± GLPG (5 mM and 0.1 μM respectively, 24 h) (C). Cell proliferation was measured after 72 h by BrdU ELISA. BrdU label was added 24 h before the assay. Viability and proliferation data are represented as percentage over control and mean ± SEM of three separate experiments. The significance was tested using one‐way anova and posthoc analysis. *p ≤ 0.05, ns = not significant.

**FIGURE 7:**
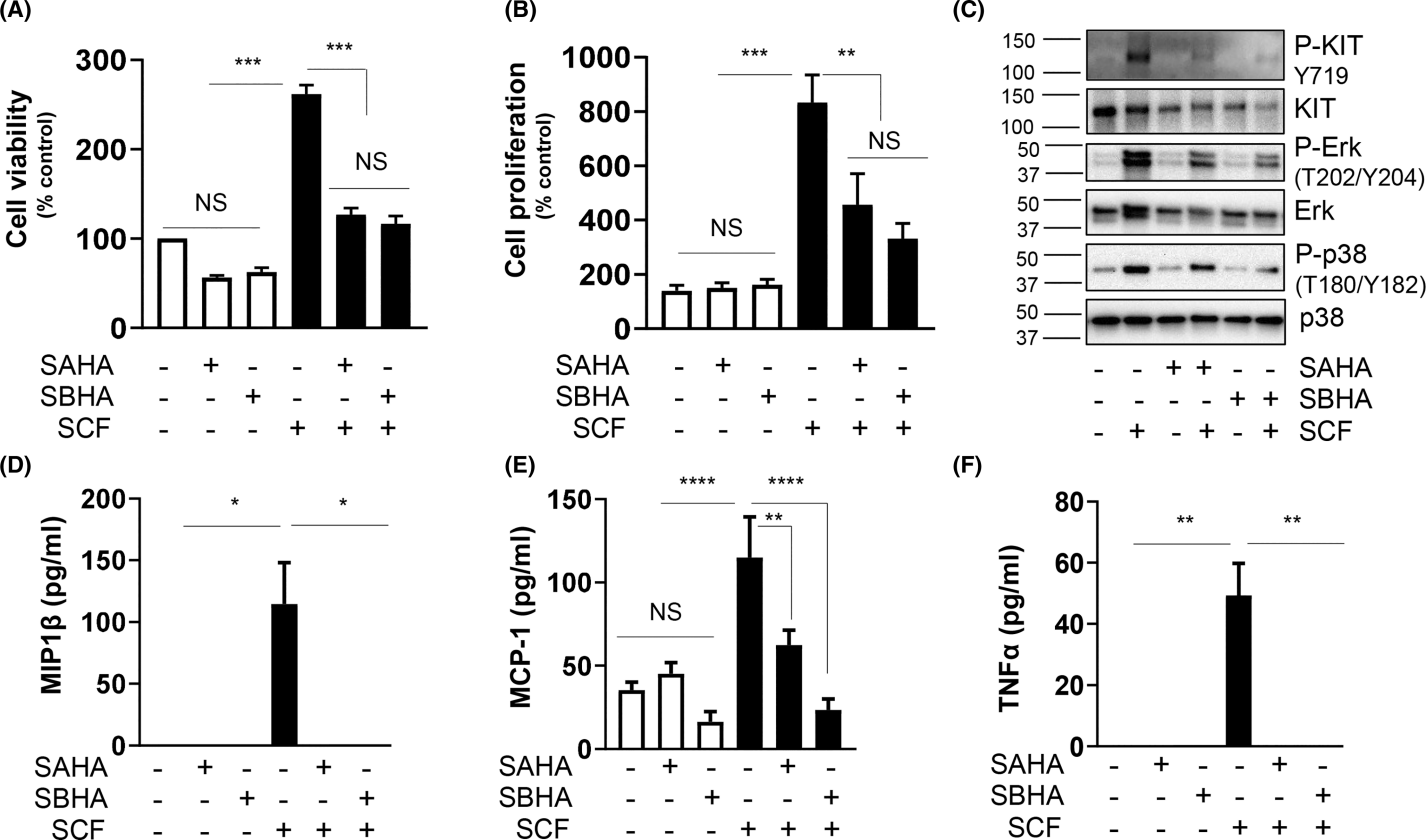
HDAC1/3 inhibitors mirror butyrate effect on SCF‐mediated MC activation. BMMCs were treated with SCF with or without and SBHA (20 μM), and SAHA (1 μM) and Cell viability was analysed by XTT assay after 72 h (A). Cell proliferation was measured after 72 h by BrdU ELISA. BrdU label was added 24 h before the assay. Viability and proliferation data are represented as percentage over control (B). BMMCs were pretreated or not with SBHA (20 μM), and SAHA (1 μM) for 24 h, and stimulated in the presence or absence of SCF (100 ng/mL) for 30 min. (C) KIT, Erk and p38 phosphorylation and expression was determined in the cell lysates by western blotting. Blots were re‐probed for total KIT and GAPDH. BMMCs were pretreated or not with butyrate (5 mM) for 24 h, and stimulated in the presence or absence of SCF (100 ng/mL) for 4 h. Culture supernatants were collected and analysed for MIP1β (D), MCP‐1 (E), TNFα (F) proteins by ELISA. Data are represented as mean ± SEM of three separate experiments. The significance was tested using one‐way anova and post hoc analysis. *p ≤ 0.05, **p ≤ 0.01, ***p ≤ 0.001, ****p ≤ 0.0001, NS = not significant.

Next, we explored the receptors through which butyrate relays its signals to attenuate MC responses. First, we analysed the expression of traditional butyrate receptors, GPR41, GPR43 and GPR109A. We found that BMMCs expressed GPR41 and GPR43, but we could not detect any GPR109A (Figure 6A). To elucidate if butyrate signals through GPR41 or GPR43, we treated BMMCs with butyrate in the presence and absence of GPR41 antagonist β‐hydroxy butyrate (BHB) and GPR43 antagonist GLPG. Neither BHB nor GLPG could rescue the inhibitory effect of butyrate on SCF‐induced MC proliferation (Figure 6B,C). Therefore, this led to the speculation that butyrate controls MC actions via epigenetic changes and *KIT* repression. In agreement, HDAC inhibitors (SAHA‐Pan HDAC inhibitor; SBHA‐ HDAC1&3 inhibitor) mirrored all the butyrate signals, including the attenuation of SCF‐mediated viability (Figure 7A), proliferation (Figure 7B), downregulation of KIT and associated signalling (Figure 7C), and mediator release (Figure 7D–F).

## 4 DISCUSSION

In the gut, bacterial fermentation of dietary fiber results in the generation of SCFA, including acetate, propionate and butyrate. SCFA are demonstrated to provide beneficial effects against autoimmune and inflammatory disorders, and they play an important role in shaping the immune system.^21^ Several studies revealed that SCFA via epigenetic modification regulate airway inflammation.^22^, ^23^, ^24^, ^25^, ^26^ Butyrate treatment (5 mM, 24 h exposure) of OVA‐sensitized guinea pig lung slices demonstrated attenuation in allergen‐induced histamine release and airway contraction.^15^ However, few studies focus on the mechanistic aspects of how SCFA affect MC function. MCs are initiators of inflammatory responses to allergens and infectious agents, and they play an important role in triggering asthma exacerbations through several inflammatory mediators.^2^, ^3^, ^4^ Understanding the regulatory signals in MC activation by SCFA is vital to therapeutically target MC‐mediated diseases, including asthma. In the present study, we demonstrated for the first time that butyrate causes butyrylation of histone H3 at lysine 9 (H3K9) in BMMCs. Further, butyrate promoted global histone acetylation and inhibited HDAC activity. In an attempt to understand the functional relevance of these changes in BMMCs, we investigated how butyrate affects the KIT receptor, as KIT is an important regulator of MC growth, differentiation, survival and chemotaxis.^27^, ^28^, ^29^ MCs are activated in an antigen (Ag)‐dependent as well as an Ag‐independent manner.^30^, ^31^, ^32^ In response to Ag stimulation, butyrate (1–25 mM) has been shown to inhibit MC degranulation.^15^, ^33^ Butyrate in murine MCs was shown to suppress FcεR1‐mediated cytokine release by inhibiting HDAC activity.^34^ Since previous studies analysed how SCFA impact MC function in response to FcεR1 activation, we focused on the Ag‐independent SCF/KIT signalling axis. We observed that butyrate treatment caused downregulation of the KIT receptor and inhibited its phosphorylation. Interestingly, apart from KIT, we also observed downregulation of FcεR1 in response to butyrate (not shown), which could also contribute to the attenuated MC mediator release by IgE reported earlier.^15^, ^33^, ^34^, ^35^ It appears that butyrate inhibits MC function by downregulating key MC receptors and associated signalling. Concurrent to KIT downregulation, we observed a significant attenuation in SCF/KIT‐mediated MC survival, proliferation, and chemokine secretion by butyrate. In accordance with our work, butyrate has been shown to inhibit cytokine transcripts in a CPII mast cell line^33^ and to inhibit MC mediator release in the gut mucosa of pigs.^35^ In an attempt to understand the mechanistic aspects of how butyrate mediates these changes downstream of KIT in BMMCs, we found that butyrate significantly attenuated SCF‐induced p38 and Erk phosphorylation. Importantly, inhibition of p38 and Erk diminished SCF‐mediated chemokine secretion. Both p38 and Erk were implicated in SCF‐mediated MC survival and proliferation.^5^ Recently, using malignant MC lines, butyrate at much lower concentrations (0.01–1 mM) was reported to suppress MC proliferation and promote differentiation via suppressing KIT.^36^ The discrepancy could be due to the differences in the MCs used (malignant MC lines vs. primary BMMCs) in both of the studies. SCFA relay their signals via cell surface G‐protein‐coupled receptors GPR41, GPR43 and GPR109a, or by acting as a HDAC inhibitor in immune cells to modulate gene expression in cell proliferation and differentiation.^37^ Interestingly, we observed that attenuation of SCF‐mediated MC function by butyrate is independent of traditional butyrate receptors. Since we observed that butyrate inhibited HDAC activity and butyrate was shown to inhibit HDACs^22^ to influence immune cell activation, we speculated that butyrate suppresses KIT expression via HDAC inhibition. In agreement, inhibition of HDAC1&3 inhibitor mimicked all of the butyrate effects observed in MCs suggesting that butyrate possibly attenuates KIT‐dependent BMMC function epigenetically via HDAC1/3 inhibition. Our results emphasize that KIT phosphorylation by SCF leads to p38 and Erk phosphorylation, followed by enhanced MC survival, proliferation, and transcriptional activation of inflammatory mediator release. Butyrate treatment via blocking HDAC1/3 causes downregulation of KIT, which in turn results in reduced KIT phosphorylation, KIT‐dependent p38 and Erk phosphorylation, MC proliferation and inflammatory mediator secretion impacting MC function. IgE‐mediated MC activation was also shown to be regulated by HDAC independent of cell surface GPCRs previously.^15^ Further, apart from MCs, SCFA at concentrations ranging from 1 to 25 mM were demonstrated to regulate survival of several immune cells, mainly via HDAC inhibition.^10^ HDAC inhibitor was also shown to attenuate food allergy responses by inhibiting IgE‐mediated MC activation.^38^ Eosinophil survival and migration were regulated by butyrate and propionate (10 mM), mimicking class IIa HDAC inhibitors.^39^ Butyrate (2 mM) was shown to attenuate lipopolysaccharide‐induced activation of human monocyte‐derived DCs.^24^

The dose of butyrate used in most in vitro studies, including ours, to determine effects on immune cell function is in low mM range, a concentration unattainable through gut microbial production. However, studies suggest that these concentrations are achievable by supplementation. Mice fed on high‐fat diets were shown to elevate circulating SCFA and were protected from allergic airway inflammation in the lung.^26^ Administration of sodium butyrate orally to mice at a dose of 5 g/kg resulted in a transient spike of butyrate in plasma to ~9 mM 15 min after dosing, and the concentration was maintained around 1 mM for 90 min after dosing.^40^ Further, similar administration of butyrate during the challenge phase resulted in decreased eosinophilia and, attenuation of Type 2 cytokines and airway hyperresponsiveness, reinforcing that low mM plasma concentrations of butyrate, even transiently by butyrate supplementation, can improve airway inflammation in vivo.^39^

Although histone acetylation traditionally is associated with increased transcriptional activation, we observed repression of the KIT and SCF‐mediated inflammatory transcripts (*MIP1β, MCP‐1, TNFα and COX‐2*) in response to butyrate. In line with our results, butyrate was shown to enhance global H3K27 acetylation, but repress IgE‐responsive genes like Bruton's tyrosine kinase, linker of activated T cells and spleen tyrosine kinase.^15^ The details of how butyrate‐mediated histone acetylation leads to transcriptional repression of KIT and other inflammatory genes needs to be investigated further. Although our current results focus on butyrate‐mediated acetylation of histones and associated SCF responses in MC, we also report significant butyrylation of histones by butyrate. Since we do not know how butyrylation impacts MC histones, or non‐histone (transcription factor) modifications by butyrate or how it contributes to MC function, it needs to be explored further.

**FIGURE 8:**
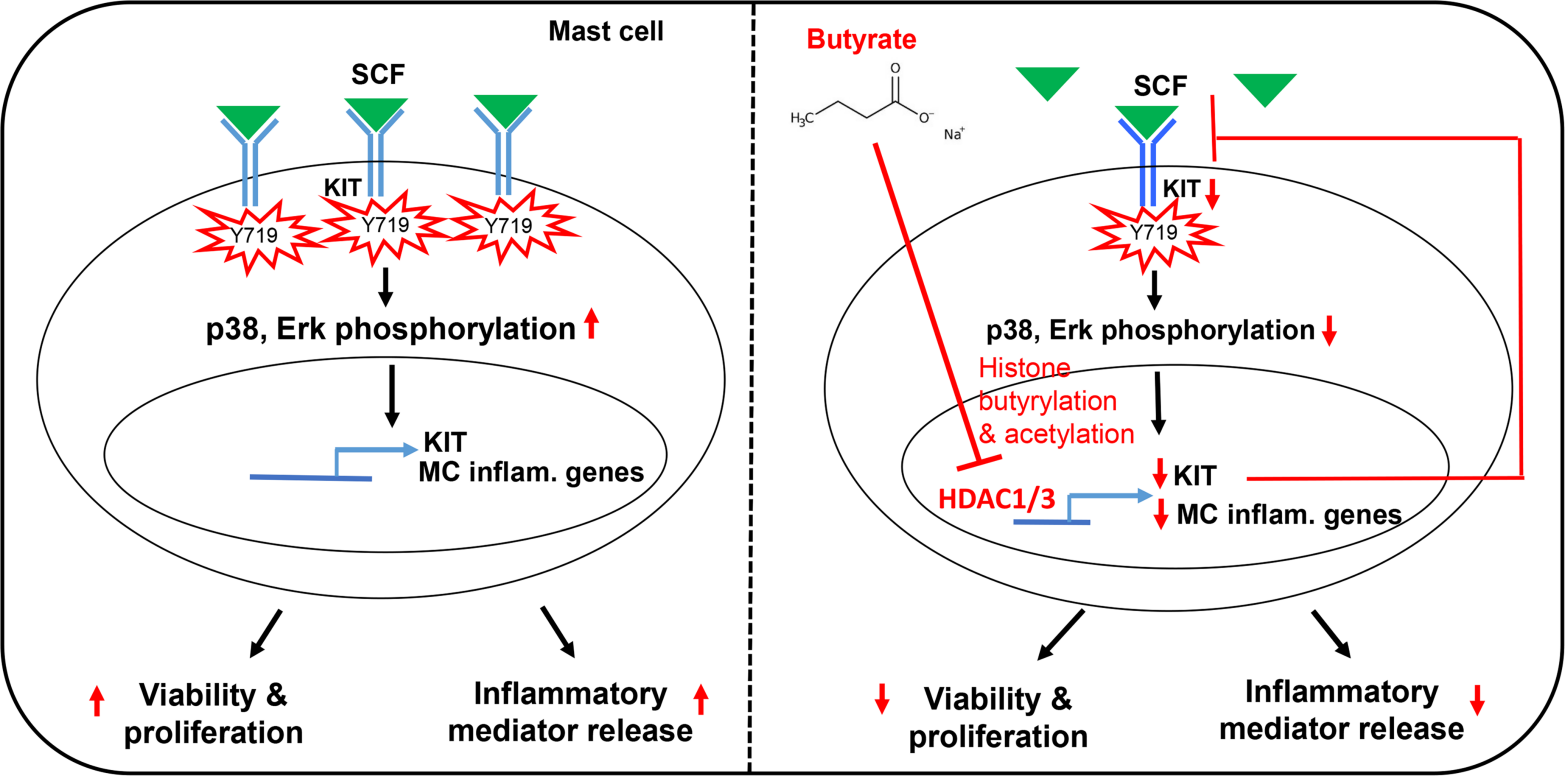
Schematic depicting putative mechanisms of epigenetic regulation of KIT and SCF‐mediated mast cell (MC) function. In untreated MC, binding of SCF to KIT induces its tyrosine phosphorylation at Y719 which in turn activates p38, Erk‐dependent expression of KIT and inflammatory genes leading to MC proliferation and secretion of inflammatory mediators. However, butyrate treatment reduces KIT expression, tyrosine phosphorylation and downstream signalling (p38 MAPK, Erk, proliferation and inflammatory mediator secretion) via epigenetic modification of histones leading to suppression of KIT‐mediated MC activation and proliferation.

In summary, we identified that butyrate epigenetically inhibits SCF‐mediated BMMC proliferation and activation by enhancing histone acetylation (Figure 8). Given that MC proliferation is increased at sites of inflammation in allergic diseases, supplementation of butyrate could offer a promising strategy in controlling MC‐mediated allergic diseases.

## AUTHOR CONTRIBUTIONS

**Ravindra Gudneppanavar:** Conceptualization (supporting); data curation (equal); formal analysis (equal); investigation (equal); methodology (equal); project administration (equal); resources (equal); software (equal); supervision (supporting); validation (equal); visualization (equal); writing – original draft (supporting); writing – review and editing (supporting). **Emma Elizabeth Sabu Kattuman:** Conceptualization (supporting); data curation (equal); formal analysis (equal); investigation (equal); methodology (equal); project administration (equal); resources (equal); software (equal); supervision (supporting); validation (equal); visualization (equal); writing – original draft (supporting); writing – review and editing (supporting). **Lakshminarayan teegala:** Conceptualization (equal); data curation (equal); formal analysis (equal); funding acquisition (supporting); investigation (equal); methodology (equal); project administration (equal); resources (equal); software (equal); supervision (equal); validation (equal); visualization (equal); writing – original draft (supporting); writing – review and editing (supporting). **Erik Southard:** Conceptualization (supporting); data curation (supporting); formal analysis (supporting); investigation (supporting); methodology (supporting); project administration (supporting); resources (supporting); software (supporting); validation (supporting); visualization (equal); writing – original draft (supporting); writing – review and editing (supporting). **Ramakumar Tummala:** Data curation (supporting); formal analysis (supporting); methodology (supporting); software (supporting); validation (supporting); writing – review and editing (supporting). **Bina Joe:** Conceptualization (supporting); methodology (supporting); resources (supporting); writing – review and editing (supporting). **Charles K Thodeti:** Conceptualization (supporting); methodology (supporting); resources (supporting); writing – original draft (supporting); writing – review and editing (supporting). **Sailaja Paruchuri:** Conceptualization (lead); data curation (supporting); formal analysis (supporting); funding acquisition (lead); project administration (lead); resources (lead); software (supporting); supervision (lead); validation (supporting); visualization (supporting); writing – original draft (lead); writing – review and editing (lead).

## CONFLICT OF INTEREST STATEMENT

Authors declare no conflict of interest.

## Data Availability

The data to support the findings of the present study are available from the corresponding author upon request.
